# Shared Decision-Making: Some cautionary observations in the context of elite sport

**DOI:** 10.1186/s40798-022-00413-2

**Published:** 2022-03-30

**Authors:** Darren James Paul, Luke Jones, Paul Read

**Affiliations:** 1 Aspetar – Qatar Orthopaedic and Sports Medicine Hospital, Research and Scientific Support, PO Box 29222, Doha, Qatar; 2 Faculty of Health Sciences, University of Hull, Hull, HU6 7RX UK; 3 Institute of Sport Exercise and Health, London, UK; 4 Division of Surgery and Interventional Science, University College London, London, UK; 5 School of Sport and Exercise Sciences, University of Gloucestershire, Gloucester, UK

**Keywords:** Athlete, Staff, Relationships, Values, Context, Mediators, Barriers

## Abstract

The concept of shared decision-making (SDM) has emerged as a key component in the return to play interface as a hallmark of good practice that is athlete focused and allows greater engagement from the athlete. SDM is an appealing, well-intentioned framework that would seemingly lend itself to effectively being implemented. However, in this editorial, we have identified concerns surrounding the social complexities of elite sports and the difficulties of truly applying this concept in practice. In what follows, we explain the dynamics associated, discuss the importance of context when considering the efficacy of this practice and lastly offer what we see as certain key issues that might impede effective SDM.

## Key Points

- Shared decision-making (SDM) has become a common theme in many practices within a professional elite sport setting. However, concerns surrounding the social complexities of elite sports mean it is difficult to truly apply in practice and is often inappropriately and/or incorrectly executed
- Practitioners should be more cognizant of the complexity associated with SDM and the individual factors that constitute an athlete’s beliefs, values and decisions as well as the potential surrounding influences in the working environment.

## Introduction

Within the highly commercialized domain of sport, the performing athletic body has become a commodity of vital importance [1]. Correspondingly, sports practitioners across the globe have rallied to devise innovative ways to train, protect, heal and improve athletes. While injuries are an inherent and unavoidable component of professional elite sports, the potential array of negative implications has manifested in large investment in the performance and rehabilitation of injured athletes. A notable development is that of the growing number of Sport Science and Medical practitioners that are actively involved in this complex and dynamic process.

Shared decision-making (SDM) is defined as a consultation process where a clinician and patient jointly participate in making a health decision, having considered the patient’s values, preferences and circumstances and discussed the options and their potential benefits and harms [2]. This approach is considered a hallmark of good clinical practice that is athlete focused and allows greater engagement. Makoul and Clayman [3] suggest SDM includes nine essential elements: problem definition; presenting and discussing options; discussing patient values and abilities; discussing provider knowledge; clarifying understanding; decision-making; and arranging follow-up. While clearly well intended and an appealing conceptual framework, this article acts as a cautionary tale that promotes critical reflection surrounding the implications associated with SDM. In what follows, we explain from a coach practitioner’s perspective the dynamics associated, discuss the importance of context when considering the efficacy of this practice and lastly offer what we see as certain key issues that might impede SDM.

## SDM in Elite Setting

Decisions about whether to return to play or continue rehabilitation when having an injury are typical of all kinds of sports [4]. Indeed, return to play decisions have to be considered as ‘risk decisions’, which are generally defined by at least one uncertain negative outcome in at least one of the alternatives [4]. The athlete, coach and healthcare practitioner serve as stakeholders in an attempt to return the athlete under the best circumstances. Specifically, the role of the athlete is to make informed preference decisions and his/her main contribution is subjective. The healthcare professional evaluates the health status of the athlete and provides objective advice on options and outcomes while a coach’s contribution relates to the sport-specific context of player availability. The healthcare professional evaluates health status and is main contribution is objective and the coach’s main contribution is to evaluate athlete’s current ability to perform within the contextual setting [2].

### Evidence-Based Practice

It is well recognized that the practitioner should use their clinical experience, along with the best research evidence and patient preferences when making decisions [5]. While SDM supports the practice of evidence-based practice (EBP), the rationale is different for each, with the former based on at least two grounds, (1) individuals may want the opportunity to be more involved and (2) and ethical autonomy that should lead to better health outcomes [6]. Despite the intended harmony, there may also be philosophical difficulties that suggest the two approaches (EBP and SDM) are not truly entwined and are fundamentally incompatible [6]. Various hierarchical models to classify the best available research have been developed and commonly depict systematic reviews and meta-analyses at the top of the pyramid, with case studies and personal opinions at the bottom. While this hierarchy may imply some sort of increasing validity and applicability, it may also emphasize that the lower sources of evidence are least preferred in practice because they require more time to identify, appraise and apply [7]. Indeed, reports show that 100% of sport scientists consider peer reviewed research as the preferred source for sport science and individualized preparation or recovery recommendations in their performance training program [8], even though this may be in conflict with the athlete’s own experience, knowledge and preferences.

### Measurement

Elwyn and colleagues (2010) said “decisions cannot be measured by reference to their outcomes” and proposed that we should emphasise “the deliberation process rather than the decision’s end results” [9]. Rather, the components of SDM, notably the outcome of a decision, readiness to make a decision, efficacy of intervention and decision aids, and decision quality should be measured in order to evaluate the effectiveness of the SDM. However, there are innate difficulties in constructing reliable tools to measure, monitor and review the implementation and efficacy of SDM for an athlete returning to play in a professional elite setting [10, 11].

Advances in technology used in the professional elite sport setting have coincided with a seemingly growing obsession in Sport Science and Medicine to measure and quantify many realms of physical, psychological, biomechanical and physiological components of an athlete. While it is clear that much of this has advanced our understanding of sports performance, what is also clear is that this process has significant implications for the management and stewardship of athletes. For example, while objective measures targeting arbitrary thresholds are promoted as a focal point for many individuals returning to play following an injury, it is not uncommon for the corresponding prescribed recovery pathways to be at odds with an athlete’s preferences, understanding of their own body, or their values. SDM does not necessarily start and end at the beginning of the rehabilitation or training cycle, nor should it be measured solely on an athlete’s successful return to play.

### Dynamic

SDM is generally viewed as a continuum, along which the extent of a patient’s or clinician’s involvement and/or responsibility for the decision-making processes varies according to the situation/injury. This seems to elicit a polarised approach that is presented at two ends of the spectrum with a start and end point, rather than a continuously modifying process. The athlete’s emotional and behavioural responses are likely to fluctuate throughout the process, from the initial diagnosis and end phase of the injury-rehabilitation and can be linked to the dynamics of the stakeholders. SDM also inherently assumes that patients are capable of taking in complex information from practitioners during a vulnerable time in their lives and then synthesizing that information to make rational treatment choices [12] in a relatively time constrained situation. Intraday variation in visceral factors, such as pain, depression, and anger, also mediate patients’ ability to make consistent treatment choices, even in the very short term, which likely contribute to athletes’ beliefs and behaviours [13]. While the premise that SDM is dynamic and complex is likely well recognised, the greater challenge is probably what practices are being considered and implemented in response to the dynamics of these changes.

## The Importance of Context

### Mediators

Despite the athlete’s preference, at the highest level of professional elite sport, there are often wider performance implications and contextual pressures that impact upon athletes [2]. Sports are primarily a social process bound by a number of contextual factors that may (in)directly and (sub)consciously have some influence on any athlete’s decision-making process; including health status, participation risk, family, teammates, management, sponsors, and supporters [14]. These mediators can have a strong influence on players and their performance, as well as their attitudes to pain and injury [15]. These social influences have shaped an athlete’s attitude towards Sports Science and Medicine practices, and practitioners as sources of authority [16], and the worth of output monitoring in general (for example, in field sports such as football it has recently been reported that some athletes disdain load monitoring practices as superfluous [17].

### Cost Benefit

An important component of SDM is to inform and engage patients about the risks and benefits of the available treatments. In a clinical setting, this seems plausible whereby the patient understands how the condition impacts upon their life and how they feel about risk [18]. In a professional elite sport setting, however, this may not be afforded since the coach ultimately decides whether an athlete is selected to compete, and there are many mediating factors that can influence these decisions. Such different roles and experience may govern the way an athlete and coach perceive situations differently [16]. In one example, emerging evidence exists that suggests a conservative approach to ACL injury is a viable option for athletes as part of their journey back to sports performance. However, there may be the general belief amongst backroom staff that the problem has not been ‘fixed’, and therefore, athletes are unlikely afforded the period of time to really consider their choices. Rather they are easily enticed into thinking that surgery allows for the rehabilitation to begin quicker and return to play sooner compared to pursuing a conservative option.

### Bias

The dynamic of the stakeholders in a professional elite sport setting is very different to that of a physician and patient in a clinical setting. Despite the best intentions to remain neutral, it is plausible that in certain circumstances whereby the situation affects possible performance, some practitioners may oppose their normal procedure [19]. Therefore, any decision may be largely influenced by the increased challenges associated with inter-relational performance pressures associated with key stakeholders and the athlete [20]. While it may often be proposed as an option, evidence indicates that the elimination of biases by simply telling patients about the existence of a particular bias and asking them not to be influenced is not actually effective [21].

While practitioners believe they are offering their athletes the autonomy, power and options by ‘involving them’ these are likely offered within an eco-system of constraints that are pinned to the club or practitioners’ ‘philosophy’ and within a bandwidth of conformity to what practitioners consider best practice. From this, it is clear there are many components of SDM that demonstrate its complex and multi-faceted nature amongst the main stakeholders (coach, practitioner and athlete) [13].

For all stakeholders to truly provide their preferences, there needs to be a non-judgmental, even playing field and where possible no agendas or conflict of interest reside. For example, the completion of the ‘exit assessment’ before returning to play is the apparent objective confirmation of being ready to return. However, evidence does suggest that athletes often return earlier and are more fearful despite being signed off ready to play [22]. Conversely, an athlete may feel ready to return before being signed off but is held back, which may call into question a patient’s ability to truly make informed choices alluding to preferences. What is more, we also argue that this very observation casts disparagement on the ability of practitioners to frame SDM as an objective, meaningful practice that they might deploy whilst claiming non-contaminated input from the athlete. It is the extreme alternative to assume that the elite athlete is ‘free’ to proffer a neutral contribution to any SDM process, let alone when said athlete finds themselves in a position of vulnerability (such as suffering a significant injury).

Anecdotal evidence shows that athletes rarely question coaches’ decisions or practices. Furthermore, coaches and athletes are likely to succumb to the social norms that can act to subconsciously influence their treatment choices. This arrangement significantly calls into question the ability of both parties to make neutral informed choices surrounding an athlete’s rehabilitation—yet for many who promote SDM this is not a consideration. The biases caused by how the information is collected and framed are always at play [23], and it is important to understand that uncertainty, error, and regret are inherent in every decision made [24].

It may be true that some athletes may be more confident or well versed in understanding analytics generated regarding their output or bodily functions and will want something closer to decision analysis—in other words, computer software that calculates the best option based on weighting probabilities with their personal utilities [6]. This circumstance is likely to be more common, particularly since analytics is so evident in all other aspects of a player’s life in professional sport; indeed, many athletes now develop in ‘data rich environments’ [25]. Indeed, in a world of increased sophistication whereby many decisions are made and greater stock is placed on data-driven decision aids (rather than solely subjective opinion), then it is reasonable to assume that many athletes are more confident and fluent in understanding the implications of analytics. Regardless, we feel that is very important that an athlete’s familiarity with analytics does not lead to the assumption that he or she should routinely be considered competent enough to fully understand the intricacies of, for example, complex medical considerations.

## The Barriers to Efficacious SDM in Practice

### Knowledge

An important component of the SDM is to provide the platform for athletes to express their preferences and feelings. There are three key steps to SDM in sports medicine; choice, option, and decision [26]. Despite its innocence, the delivery of choices may be primed in a way that makes it fraught with challenges and bias and delivered in a way that is void of all necessary knowledge and based on an incomprehensive base of information. Conversely, providing many options increases the complexity of the decision and risks creating greater decision uncertainty for patients [27]. Indeed, is the athlete’s choice truly based on their underpinning beliefs and education and if so, what happens if this severely opposes that of the medical team and higher management, or indeed is at odds with the position of the team/stage of the season, etc.?

The key feature of SDM is that it promotes a capacity to arrive at a well-informed choice and as a result of a largely uncoerced joint decision. While this may be the narrative that is promoted regarding the perceived positive aspect of SDM, more often than not it is likely that this does not happen. While some athletes may indeed possess innate qualities (e.g. good common and specific knowledge, good life skills and experience, a real willingness to learn and ask questions and engage in conversations) that allow them to make well-educated decisions; likewise, there are also many that may not even be able to actually comprehend the choices, options and decisions available before them (for example as a result of their level of education or age). Would a detailed open dialogue be afforded to all athletes, or could we expect practitioners approach a young athlete and seasoned professional in a different way and when, or if, does this change in an athlete’s career? Is it simply when the athlete potentially becomes more vocal after previous experience? In this way, we argue here that SDM opens up a whole host of questions and variables that require far more than cursory acknowledgement, and indeed, much greater attention.

### Power

A large premise of SDM is to give ownership and control to the athlete. While this may sound theoretically appealing, sport is oriented to the production and management of athletes so that they infrequently question institutional norms [28]. Indeed, it may be difficult to distinguish between an athlete centred approach that encompasses ‘buy in’ and ‘engagement’ and that of a subtle imposition of ‘disciplinary power’ upon the athlete [29, 30]. Arguably, Sports Science and Medicine are central to the process of the imposition of disciplinary power as part of the unquestioned day-to-day regularities of ‘normality’ for an athlete [28]. For example, during the course of a day, an athlete will have had many exposures to coaching and Sport Science Medicine staff and been requested to perform many duties with which they are expected to dutifully comply [31]. This arrangement procures their obedience to workplace norms (including expectations regarding the normalized management of pain and injury, as well as training load). Examples include an acceptance of what tactics to conform to, physical effort/outputs they must achieve, supplements they must consume, testing measures they must complete and the thresholds they need to attain, as well as the fines that may be sanctioned in line with the club’s code of conduct, to name just a few. Athletes often conform to their sanctioned roles because “it would feel very uncomfortable if I were in a position where I felt like I were challenging the doctor/practitioner and his authority.”, and because of the precarious nature of playing/performance positions in elite sport institutions [32]. Although the conversational activities may at times suggest something of a process of co-opting patients to act ‘responsibly’ and through their own choice, the deployment of other discursive strategies makes it hard not to view the coach and practitioner contributions as a level of paternalism [33], that importantly, often times serves to render the athlete as silent rather than as empowered to legitimately proffer their own unbiased view. Conversely, athletes in certain sports may actively select practitioners that do not challenge their beliefs/desires which at times may make athletes more vulnerable in the SDM process. Our point therefore is that the implementation of SDM in a professional elite sport setting without careful consideration of the way athletes are positioned by their working roles may be at worst futile, and at best insubstantial as long as the coercive, normalized relations of power common to professional elite sports spaces remain ignored [34].

## Conclusion

SDM is an appealing and well-intended framework that would ideally lend itself to being effective in its implementation. However, in this editorial, we have identified concerns surrounding the social complexities of elite sports and the difficulties of truly applying this concept in practice. As a result, we also caution that SDM can often be inappropriately and/or incorrectly executed specifically during the rehabilitation process. There is a difference between intention and perception of SDM and reality and its execution. We argue that consideration for the deployment of SDM will automatically bring benefit to the rehabilitation process without paying close attention to the significant social pressures that abound in professional elite sports contexts might be considered a little naïve. Simply providing lip service to ‘implementing and using SDM’ based on the premise that the athlete was present during certain discussions is likely an oversimplification of the issues at hand. Therefore, despite the best intentions by coaches, and sport science and medical staff to propagate SDM in a sports setting, practitioners would be better positioned to circumvent its complexity and acknowledge that its theoretical intent unlikely easily manifests itself in practice.

## Data Availability

Not applicable.

## Abbreviations

SDM: Shared decision-making
EBP: Evidence-based practice
ACL: Anterior cruciate ligament
